# Hypoalbuminemia and obesity class II are reliable predictors of peri-prosthetic joint infection in patient undergoing elective total knee arthroplasty

**DOI:** 10.1186/s43019-020-00040-9

**Published:** 2020-05-11

**Authors:** Sheryl Lok-Chi Man, Wai-Wang Chau, Kwong-Yin Chung, Kevin Ki Wai Ho

**Affiliations:** Department of Orthopaedics and Traumatology, Chinese University of Hong Kong, Prince of Wales Hospital, Shatin, Hong Kong SAR

**Keywords:** Elective total knee arthroplasty, Nutrition, Obesity, Serum albumin, Total lymphocyte count

## Abstract

**Background:**

Malnutrition is a common and modifiable risk factor for postoperative complications and adverse outcomes in orthopedics. The purpose of this study was to identify biomarkers of malnutrition in patients undergoing elective total knee arthroplasty (TKA) that are predictive of adverse in-hospital postoperative complications, to facilitate the identification of at-risk patients for nutritional optimization before surgery.

**Methods:**

A total of 624 patients who underwent elective TKA between 2013 and 2017 were evaluated; potential biomarkers of preoperative malnutrition, including hypoalbuminemia (serum albumin < 3.5 g/dL), total lymphocyte count (TLC < 1500 cells/mm^3^), and body mass index (BMI), were assessed for any association with in-hospital postoperative complications.

**Results:**

The prevalence of hypoalbuminemia, low TLC, overweight, obesity class I, and obesity class II were, respectively 2.72%, 33.4%, 14.8%, 44.5%, and 26.9%. There was a significant association between hypoalbuminemia and obesity class II (BMI ≥ 30.0 kg/m^2^) with rates of peri-prosthetic joint infection, and no significant association between such complications and low TLC, overweight, or obesity class I. Logistic regression analysis showed that patients with hypoalbuminemia or being in obesity class II with gouty arthritis were more likely to suffer from peri-prosthetic joint infection.

**Conclusions:**

Hypoalbuminemia and obesity class II together is a reliable biomarker of preoperative malnutrition for predicting peri-prosthetic joint infection after elective TKA, whereas low TLC, overweight, and obesity class I were not significantly associated with an increased risk of such complications.

## Background

Malnutrition has long been linked to postoperative complications and adverse outcomes in a variety of surgical fields [1], such as increased susceptibility to infection [2, 3], delayed wound healing [4, 5], and an increased frequency of decubitus ulcers. In particular, it is a modifiable risk factor, as evidenced by studies that have associated optimization of preoperative nutrition with improved surgical outcomes [6, 7]. Therefore, it is important to identify those patients who are at risk so that appropriate nutritional support can be implemented.

A range of methods for nutritional status assessment has been proposed; a comprehensive assessment may include measurements of dietary intake, clinical assessment, anthropometric measurements, and biochemical measurements of serum protein, micronutrients and metabolic parameters [8]. Many of the signs of malnutrition, however, only manifest in extreme cases. Thus, it is crucial to identify sensitive markers that can be utilized to screen for clinical as well as subclinical malnutrition patients.

In orthopedic patients, the prevalence of clinical and subclinical malnutrition has been reported to be up to 42.4% [9]. Common markers of malnutrition that have been studied include low serum albumin as a marker of protein status, low total lymphocyte count (TLC), and an excessively high or low body mass index (BMI). They have been compared against various adverse surgical outcomes, including peri-prosthetic joint infection such as deep surgical site infection (SSI) or implant infection [10–12], delayed wound healing [4, 5, 13], unplanned intubation, and intensive care unit (ICU) admission [11], postoperative anemia and cardiac complications [14], and length of hospital stay [15, 16]. However, conflicting results have been reported; for example, while hypoalbuminemia (serum albumin < 3.5 g/dL) has been associated with an increased risk of peri-prosthetic infection [10, 11] and longer than average hospital stay [15, 16] its effect on wound healing is less clear – Marín et al. (2002) [13] reported no significant predictive value of hypoalbuminemia on wound healing, yet Greene et al. (1991) [5] reported a five-fold increase in the frequency of major wound complications.

The primary purpose of this analysis was to identify biomarkers of malnutrition in patients undergoing elective total knee arthroplasty (TKA) that are predictive of adverse in-hospital postoperative complications, which would facilitate the identification of at-risk patients for nutritional optimization before surgery. This was the first of such analyses in the Asian-Pacific region and could provide insight into the relationship between malnutrition and post-TKA complications in this population.

## Methods

### Design and participants

In this analysis, patients who underwent elective TKA between 2013 and 2017 in Prince of Wales Hospital in Hong Kong were identified using the electronic Clinical Management System (CMS); 624 patients were identified, and their data reviewed. In our clinic, all patients who underwent elective TKA needed to be diabetic controlled and maintain a normal hemoglobin level before surgery. As a results, all recruited patients were neither hyperglycemic nor anemic. Patients’ sex, age, height, weight, and diagnosed comorbidity based on the Charlson Comorbidity Index [17], with modifications made for this orthopedic study, were recorded.

The subjects were stratified into being with or without hypoalbuminemia (serum albumin < 3.5 g/dL), with or without low TLC (TLC < 1500 cells/mm^3^), and normal weight or underweight (BMI < 18.5 kg/m^2^), overweight (BMI 23.0–24.9 kg/m^2^), obesity I (BMI 25.0–29.9 kg/m^2^) and obesity II (BMI ≥ 30.0 kg/m^2^) groups. Of note, the BMI cut-off value for various groups in the Asia-Pacific population [18] is lower than that of the World Health Organization (WHO) international cut-off.

### Outcome measurement

In-hospital postoperative complications were defined and classified into surgical-related, or anesthesia- or medical-status-related. Surgical-related complications analyzed were peri-prosthetic joint infection including deep SSI and implant infection, hematoma requiring drainage or pseudoaneurysm thrombosis at the surgical site, and wound complications; anesthesia- or medical-status-related complications included systemic infection, intensive care unit (ICU) admission, cerebrovascular accident, acute renal failure, deep vein thrombosis (DVT), pulmonary embolism (PE), and cardiovascular complications (Table 1) [19, 20]. Minor or unrelated complications excluded are intraoperative technical complications, wound pain or erythema, skin blisters, non-infective wound discharge, acute retention of urine, transient fever without identifiable source, transient delirium, asthma attack, gouty attack, hypercalcemia, gastric ulcer, and late complications that occurred after discharge such as hypertrophic scar formation.

**Table 1:** In-hospital postoperative surgical-related and anesthesia/medical-status-related complications

Complications	Examples
Surgical-related:	
Peri-prosthetic joint infection	Deep surgical site infection
	Implant infection
Hematoma requiring drainage/pseudoaneurysm thrombosis	
Wound complications	Infection
	Dehiscence
	Necrosis requiring debridement
Anesthesia/medical-status-related:	
Systemic infection	Urinary tract infection or pyelonephritis
	Pneumonia
Intensive care unit (ICU) admission	
Cerebrovascular accident	Stroke
	Transient ischemic attack
Acute renal failure	
Deep vein thrombosis (DVT)	
Pulmonary embolism (PE)	
Cardiovascular complications	Arrhythmias
	Acute coronary syndrome
	Heart failure

### Statistical analysis

Statistical analysis was performed to identify any malnutrition biomarkers that are predictive of potential adverse in-hospital postoperative complications after elective TKA. The prevalence of preoperative comorbidities in low TLC, hypoalbuminemia, and obesity groups were compared and analyzed. Chi-square tests were used to examine the correlation between various comorbidities and the 10 postoperative complications. Further analyses with logistic regression modeling were carried out for any comorbidities that were significantly correlated to postoperative complications. All *P* values < 0.05 were deemed statistically significant. All statistical analysis was performed using IBM SPSS version 25 (Armonk, NY, USA).

## Results

### Descriptive statistics of patients in hypoalbuminemia, low TLC, and obesity groups

Prevalence, demographics, and comorbidities of patients in the hypoalbuminemia, low TLC, and the five BMI groups were summarized in Table 2. Six hundred and twenty-four patients with preoperative albumin level data were identified, of which the prevalence of hypoalbuminemia (serum albumin < 3.5 g/dL) is 2.7%. Five hundred and forty-two patients with TLC data were identified (86.6% of the cases reviewed), of which the prevalence of low TLC (TLC < 1500 cells/mm^3^) is 33.4%. A total of 433 patients with available preoperative BMI were analyzed (68.8% of the cases reviewed). They were categorized into five groups – normal weight, underweight (BMI < 18.5 kg/m^2^), overweight (BMI 23.0–24.9 kg/m^2^), obesity I (BMI 25.0–29.9 kg/m^2^), and obesity II (BMI ≥ 30.0 kg/m^2^); the respective prevalence of each of the groups were 12.5%, 1.2%, 14.8%, 44.5%, and 26.9%. The mean BMI was 27.5 kg/m^2^.

**Table 2:** Preoperative albumin level, total lymphocyte count (TLC), and body mass index (BMI)* analyses – Patient demographics and clinical characteristics

Demographic variables	Normal albumin level (*N* = 607)	Hypoalbuminemia^a^ (*N* = 17)	Normal TLC (*N* = 361)	Low TLC^b^ (*N* = 181)	Normal weight^c^ (*N* = 54)	Under-weight^d^ (*N* = 5)	Overweight^e^ (*N* = 64)	Obesity I^f^ (*N* = 192)	Obesity II^g^ (*N* = 116)
Age (mean ± SD)	67.3 ± 7.2	68.9 ± 8.2	67.2 ± 7.4	68.1 ± 6.9	67.8 ± 7.6	57.6 ± 6.3	69.1 ± 8.1	68.8 ± 7.2	64.8 ± 6.0
Sex (%)									
Female	75.5	70.6	76.2	71.3	68.5	80.0	73.4	82.3	76.7
Male	24.6	29.4	23.8	28.7	31.5	20.0	26.6	17.7	23.3
Comorbidities (% (*N*))									
Hypertension	69.9 (424)	88.2 (15)	68.1 (246)	72.4 (131)	64.8 (35)	40.0 (2)	62.5 (40)	72.4 (139)	73.3 (85)
Diabetes mellitus	26.0 (158)	29.4 (5)	26.6 (96)	22.7 (41)	22.2 (12)	0.0 (0)	18.8 (12)	25.5 (49)	26.7 (31)
Renal disease	3.1 (19)	23.5 (4)	2.8 (10)	5.0 (9)	1.9 (1)	0.0 (0)	3.1 (2)	4.7 (9)	6.0 (7)
Stroke/TIA	5.1 (31)	5.9 (1)	6.4 (23)	3.3 (6)	9.3 (5)	0.0 (0)	7.8 (5)	6.9 (13)	3.4 (4)
Rheumatological disease	4.4 (27)	17.6 (3)	3.3 (12)	8.3 (15)	11.1 (6)	100.0 (5)	7.8 (5)	2.1 (4)	0.9 (1)
Gouty arthritis	3.1 (19)	17.6 (3)	2.5 (9)	5.5 (10)	0.0 (0)	0.0 (0)	3.1 (2)	5.7 (11)	5.2 (6)
Chronic liver disease	1.2 (7)	0.0 (0)	0.8 (3)	1.7 (3)	0.0 (0)	0.0 (0)	3.1 (2)	1.0 (2)	1.7 (2)
Hypothyroidism	3.8 (23)	0.0 (0)	4.4 (16)	2.2 (4)	1.9 (1)	0.0 (0)	3.1 (2)	4.2 (8)	8.6 (10)
Depression	2.5 (15)	11.8 (2)	2.8 (10)	2.2 (4)	1.9 (1)	0.0 (0)	0.0 (0)	3.6 (7)	3.4 (4)
Psychosis	0.3 (2)	0.0 (0)	0.6 (2)	0.0 (0)	0.0 (0)	0.0 (0)	0.0 (0)	0.5 (1)	0.0 (0)
History of DVT or PE	2.1 (13)	0.0 (0)	2.5 (9)	1.1 (2)	1.9 (1)	0.0 (0)	0.0 (0)	1.0 (2)	0.9 (1)
Chronic pulmonary disease	0.7 (4)	0.0 (0)	1.1 (4)	0.0 (0)	0.0 (0)	0.0 (0)	0.0 (0)	1.6 (3)	0.0 (0)
History of myocardial infarct	5.3 (32)	0.0 (0)	4.7 (17)	7.2 (13)	11.1 (6)	0.0 (0)	7.8 (5)	4.2 (8)	5.2 (6)
Congestive heart failure	0.5 (3)	0.0 (0)	0.6 (2)	0.6 (1)	0.0 (0)	0.0 (0)	0.0 (0)	0.0 (0)	0.9 (1)
Arrhythmia	3.1 (19)	0.0 (0)	3.3 (12)	3.3 (6)	7.4 (4)	0.0 (0)	6.3 (4)	1.6 (3)	1.7 (2)
Valvular disease	0.8 (5)	0.0 (0)	0.8 (3)	0.6 (1)	0.0 (0)	0.0 (0)	1.6 (1)	0.5 (1)	2.6 (3)
Peptic ulcer disease	0.5 (3)	11.8 (2)	0.3 (1)	0.6 (1)	0.0 (0)	0.0 (0)	0.0 (0)	1.6 (3)	0.0 (0)
Any malignancy	4.1 (25)	0.0 (0)	5.0 (18)	4.4 (8)	1.9 (1)	0.0 (0)	3.1 (2)	8.3 (16)	3.4 (4)

Key: *DVT* deep vein thrombosis, *PE* pulmonary embolism, *SD* standard deviation, *TIA* transient ischemic attack

*BMI cut-off values for the Asia-Pacific population with reference to the World Health Organization (WHO) international standard [18]

^a^Hypoalbuminemia defined as preoperative serum albumin < 3.5 g/dL

^b^Low TLC defined as TLC < 1500 cells/mm^3^

^c^Normal weight defined as BMI

^d^Underweight defined as BMI < 18.5 kg/m^2^

^e^Overweight defined as BMI 23.0–24.9 kg/m^2^

^f^Obesity I defined as BMI 25.0–29.9 kg/m^2^

^g^Obesity II defined as BMI ≥ 30.0 kg/m^2^

### Correlation between comorbidities and postoperative complications

Correlations between various operative comorbidities and postoperative complications were made using chi-square tests (Tables 3 and 4). It demonstrated that hypoalbuminemia and obesity class II correlate with a significantly higher risk of peri-prosthetic joint infection (Table 3, comparisons with significant differences were bolded).

**Table 3:** Correlations between preoperative comorbidities and postoperative surgical-related complications

Preoperative comorbidities	Peri-prosthetic joint infection		Hematoma requiring drainage/Pseudoaneurysm thrombosis		Wound complications	
	Yes	No	Yes	No	Yes	No
Low total lymphocyte count (TLC)						
Yes	1 (25.0)	180 (33.5)	0	181 (33.5)	1 (12.5)	180 (33.7)
No	3 (75.0)	358 (66.5)	1 (100.0)	360 (66.5)	7 (87.5)	354 (66.3)
Hypoalbuminemia						
Yes	**2 (40.0)**	**15 (2.4)**	0	17 (2.7)	0	17 (2.8)
No	**3 (60.0)**	**604 (97.6)**	2 (100.0)	605 (97.3)	9 (100.0)	598 (97.2)
BMI						
Underweight	**0**	**7 (1.6)**	0	7 (1.6)	0	7 (1.6)
Normal	**0**	**54 (12.6)**	0	54 (12.5)	0	54 (12.6)
Overweight	**0**	**64 (14.9)**	0	64 (14.8)	1 (16.7)	63 (14.8)
Obesity I	**0**	**192 (44.8)**	1 (100.0)	191 (44.2)	1 (16.7)	191 (44.7)
Obesity II	**4 (100.0)**	**112 (26.1)**	0	116 (26.9)	4 (66.7)	112 (26.2)

Keys:

Numbers in bold: comparisons with statistical significances (*p* < 0.05)

Low total lymphocyte count (TLC) defined as TLC < 1500 cells/mm^3^

Hypoalbuminemia defined as preoperative serum albumin < 3.5 g/dL

**Table 4:** Correlations between preoperative comorbidities and postoperative anesthesia/medical-status-related complications

Preoperative comorbidities	Systemic infection		ICU admission		Cerebrovascular accident		Acute renal failure		Deep vein thrombosis		Pulmonary embolism		Cardiovascular complications	
	Yes	No	Yes	No	Yes	No	Yes	No	Yes	No	Yes	No	Yes	No
Low total lymphocyte count (TLC)														
Yes	6 (35.3)	175 (33.3)	1 (100.0)	180 (33.3)	0	180 (33.3)	0	180 (33.3)	6 (40.0)	175 (33.2)	1 (50.0)	180 (33.3)	0	181 (33.7)
No	11 (64.7)	350 (66.7)	0	361 (66.7)	0	361 (66.7)	0	361 (66.7)	9 (60.0)	352 (66.8)	1 (50.0)	360 (66.7)	5 (100.0)	356 (66.3)
Hypoalbuminemia														
Yes	0	17 (2.8)	0	17 (2.7)	0	17 (2.7)	0	17 (2.7)	0	17 (2.8)	0	17 (2.7)	0	17 (2.8)
No	17 (100.0)	590 (97.2)	1 (100.0)	606 (97.3)	0	606 (97.3)	1 (100.0)	606 (97.3)	16 (100.0)	591 (97.2)	2 (100.0)	605 (97.3)	7 (100.0)	600 (97.2)
BMI														
Underweight	0	7 (1.7)	0	7 (1.6)	0	7 (1.6)	0	7 (1.6)	0	7 (1.7)	0	7 (1.6)	0	7 (1.6)
Normal	2 (13.3)	52 (12.4)	0	54 (12.5)	0	54 (12.5)	0	54 (12.5)	0	54 (12.8)	0	54 (12.5)	1 (25.0)	53 (12.4)
Overweight	1 (6.7)	63 (15.1)	0	64 (14.8)	0	64 (14.8)	0	64 (14.8)	3 (30.0)	61 (14.4)	0	64 (14.8)	1 (25.0)	63 (14.7)
Obesity I	7 (46.7)	185 (44.3)	0	192 (44.3)	0	192 (44.3)	0	192 (44.3)	4 (40.0)	188 (44.4)	0	192 (44.4)	1 (25.0)	191 (44.5)
Obesity II	5 (33.3)	111 (26.6)	0	116 (26.8)	0	116 (26.8)	0	116 (26.8)	3 (30.0)	113 (26.7)	1 (100.0)	115 (26.6)	1 (25.0)	115 (26.8)

Keys:

Low TLC defined as TLC < 1500 cells/mm^3^

Hypoalbuminemia defined as preoperative serum albumin < 3.5 g/dL

*ICU* intensive care unit

### Logistic regression analysis on postoperative complications

Logistic regression models of peri-prosthetic joint infection on hypoalbuminemia and various obesity groups were carried out (Tables 5 and 6). The results demonstrated that hypoalbuminemic patients with gouty arthritis were 16.28 times more likely to have peri-prosthetic joint infection after elective TKR (*r*^2^ = 0.14, *P* = 0.03) (Table 5). Moreover, obesity II patients with gouty arthritis were 27.00 times more likely to have peri-prosthetic joint infection after TKR (*r*^2^ = 0.23, *P* < 0.01) (Table 6).

**Table 5:** Logistic regression models on hypoalbuminemia

	Yes (albumin < 3.5 g/dL)				No (albumin ≥ 3.5 g/dL)			
	*r*^2^	B ± SE	Odds ratio (95% CI)	*P* value	*r*^2^	B ± SE	Odds ratio (95% CI)	*P* value
*Peri-prosthetic joint infection*								
Gouty arthritis	0.14	2.79 ± 1.25	16.28 (1.41, 187.85)	0.03	0.09	1.87 ± 1.61	6.50 (0.28, 151.12)	0.24

Legend: *B* regression coefficient, *CI* confidence interval, *SE* standard error

**Table 6:** Logistic regression models on body mass index (BMI)

	Normal (*N* = 54)		Overweight (*N* = 64)		Obesity I (*N* = 192)		Obesity II (*N* = 116)	
	Odds ratio (95% CI)	*P* value	Odds ratio (95% CI)	*P* value	Odds ratio (95% CI)	*P* value	Odds ratio (95% CI)	*P* value
*Peri-prosthetic joint infection*								
Gouty arthritis	–	NS	–	NS	–	NS	27.00 (2.99, 243.53)	0.003

Legend: *B* regression coefficient, *CI* confidence interval, *NS* no statistical significance, *SE* standard error

## Discussion

Malnutrition can be defined and measured in a number of ways, including serological laboratory values, anthropometric measurements, and standardized scoring tools such as the Rainey-MacDonald Nutritional Index [21] and the Mini Nutritional Assessment [22]. In relation to biomarkers for prediction of postoperative complications in orthopedics, the most accessible and commonly used markers are serum albumin level < 3.5 g/dL, TLC < 1500 cells/mm^3^, and BMI, which were studied in this analysis.

This study reviewed 624 patients who underwent TKA in the Prince of Wales Hospital in Hong Kong between 2013 and 2017 and evaluated the association between biomarkers of preoperative nutritional status and in-hospital postoperative complications. Although there were studies in Asia [23–26] that looked into the association between obesity and various post-TKA outcomes, and a large-scale Korean study [27] on malnutrition and post-TKA wound complications, none provided a comprehensive evaluation of the relationship between malnutrition and postoperative complications; as the first of such analyses in the Asia-Pacific region, this study provided new insights into this topic.

This study demonstrated a significant association between hypoalbuminemia and obesity class II with increased the risk of peri-prosthetic joint infection. No such association was identified in patient with low TLC, overweight, or obesity class I.

### Obesity

The term “malnutrition” distinguishes itself from “nutrient deficiency” by encompassing both deficiencies and excess in nutrients, and obesity is a form of the latter with caloric overnourishment. It is associated with a host of morbidities, especially metabolic and cardiovascular risks, and in orthopedics, increased incidence of osteoarthritis that may ultimately lead to the need for TKA. Interestingly, recent evidence has shown that increasing BMI is paradoxically associated with malnutrition in terms of micronutrient and protein deficiencies [28, 29], which may be one of the mechanisms by which obesity increases risks of postoperative morbidity and mortality, but this topic is beyond the scope of this study.

Although it is largely agreed that obesity is associated with an increased in the incidence of postoperative complications in total joint arthroplasty, studies have shown somewhat conflicting findings. Suleiman et al. (2012) [30] analyzed 1731 cases, and demonstrated no statistical difference in peri-operative complication rates in patients undergoing TKA or total hip arthroplasty (THA) across various BMI categories. However, Namba et al. (2005) [31] reported that patients with BMI ≥ 35 kg/m^2^ had 6.7 times higher rates of postoperative infection (*N* = 1813), but no significant association with DVT, cardiac, gastrointestinal, or genitourinary complications. Similarly, Miric et al*.* (2002) [32] found an increased risk of one or multiple postoperative complication in obese patients (BMI ≥ 35 kg/m^2^), yet no significant difference in the rates of specific complications compared to non-obese patients. On the other hand, some studies found associations with postoperative complications only in cases of more severe obesity – the study by Fu et al*.* (2016) [28] of 71,599 TKA cases showed that only patients with BMI ≥ 40 kg/m^2^ were associated with any postoperative complications, wound complications, and the need for reoperation. Malinzak et al*.* (2009) [33] reported an increased risk of peri-prosthetic infection (odds ratio 21.3) in patient with BMI > 50 kg/m^2^. However, it should also be noted that extreme obesity is uncommon in this locality; in fact, there were only two cases with BMI > 40 kg/m^2^ in this study. Consequently, this discrepancy in the demographics may result in difference between the risks of postoperative complications in the Asia-Pacific and Western populations.

All in all, although meta-analysis is not available in the literature, many studies had shown that obesity is associated with an overall increased risk of postoperative complications, but not with any specific complication except infection and thromboembolic events. This is consistent with the results of this analysis, that patients with BMI ≥ 30 kg/m^2^ suffered from an increased risk of peri-prosthetic joint infection. However, it must be noted that the cut-off for obesity II was at BMI ≥ 30 kg/m^2^ due to the study population being Asian in ethnicity, as contrast to the WHO standard of ≥ 35 kg/m^2^ that is used more commonly for Caucasian populations. In addition, due to the difference in metabolism and fat distribution, an Asian-Pacific patient with the same BMI as a Caucasian patient had a higher risk of comorbidity [18]. This may explain why this study was able to demonstrate an increased risk of post-TKA complication at a lower BMI compared to other studies.

The association between obesity and post-TKA complications is of particular interest because the prevalence of obesity is particularly high in elective TKA; in various series, the prevalence ranged from 52 to 61.8% [28, 31]. In this analysis, the prevalence of obesity (BMI ≥ 25 kg/m^2^) was 86.2%, with a mean BMI of 27.5 kg/m^2^. Although the precise reason for this higher prevalence of obesity in our study population is beyond the scope of this analysis, possible reasons include the long waiting list for elective TKA in public hospitals in Hong Kong leading to patients undergoing the surgery at a more advanced stage of disease, and selection bias as obese patients tend to be more symptomatic comparing to non-obese patients and, thus, are more often indicated for TKA.

### Hypoalbuminemia

Serum albumin as a biomarker of protein status and malnutrition had been intensively investigated. It had been used to detect acute nutritional change by virtue of its short half-life, and had been shown to be a sensitive indicator of marginal nutrient deficiency [5, 9]. Combined with the ease of access to its serological level, and the potential for hypoalbuminemia to be corrected preoperatively in elective surgeries like TKA, it has been a popular biomarker in the orthopedic literature.

The association between hypoalbuminemia (commonly defined as serum albumin < 3.5 g/dL) and various post-TKA complications, however, remains controversial despite numerous studies; it appears to be associated with some complications but not others. A meta-analysis by Yuwen et al. (2017) [10] included 112,183 cases from 13 studies, and revealed a 2.5-fold increase in risk of SSI in patients with hypoalbuminemia. The analysis by Kamath et al. (2017) of 4551 cases [11] not only supported the association with SSI, but also increased risk of unplanned intubation, intraoperative or postoperative blood transfusion, acute renal failure, and mortality and coma. Hypoalbuminemia has also been associated with a longer than average hospital stay by Lavernia et al. (1990) [15] and Dreblow et al. (1981) [16]. Its relationship with wound healing, however, is less clear – studies by Marín et al. (2002) [13] and Morey et al. (2016) [27] of, respectively, 170 and 3169 patients, reported no significant predictive value of hypoalbuminemia on wound healing, yet Greene et al. (1991) [5] reported a five-fold increase in the frequency of major wound complication. Further investigations and meta-analysis would be essential for the establishment of association between hypoalbuminemia and various post-TKA complications.

Of note, the prevalence of hypoalbuminemia in this series was only 2.72%, limiting the statistical power of this study to demonstrate any association or the lack thereof. Despite the significantly higher incidence of peri-prosthetic joint infection in the hypoalbuminemia group, it is difficult to draw any meaningful conclusion in view of the small sample size and limited statistical power. The prevalence of hypoalbuminemia (as defined as < 3.5 g/dL) is highly variable in the literature, ranging from 4.0 to 15% [11, 33]. The prevalence of hypoalbuminemia in this study was at the lower end of this spectrum, possibly reflecting the fact that protein deficiency is less common in this locality; in fact, a study by Woo et al. [34] on the protein nutritional status of elderly people in Hong Kong found that their mean protein intake was well above the WHO recommendation, and protein nutritional status, as measured by various biomarkers including serum albumin, appeared to be adequate amongst the elderly Chinese living in the community. Therefore, the study of hypoalbuminemia by locality likely require a larger sample size.

### TLC

TLC was less intensively studied comparing to serum albumin as a biomarker for malnutrition. A low TLC (commonly defined as < 1500 cells/mm^3^) is thought to be associated with the impaired immunity to prevent or eradicate postoperative infection and wound healing [29]. The literature in other areas of orthopedics reported conflicting results; studies on spinal surgery reported low TLC to be associated with higher rates of postoperative infection and poor wound healing [35], and a normal TLC with lower rates of overall infection [36]. However, a retrospective, multicenter, case-control study found no statistically significant association between low TLC and risk of infection [37]. Even fewer studies focused on low TLC and total joint arthroplasty. The analysis by Marín et al. (2002) [13] of 170 patients undergoing elective TKA reported a three-times higher rate of wound complications in patient with low TLC, but a study of 3169 cases by Morey et al. (2016) [27] reported no association between low TLC and functional outcomes or incidence of wound complication. These conflicting results calls for further investigations into the accuracy and reliability of TLC as a surrogate marker of malnutrition in predicting postoperative complications in various fields of orthopedic surgery.

### Logistic regression analysis – gouty arthritis

Logistic regression analysis demonstrated that patients with gouty arthritis along with hypoalbuminemia or those who are in obesity class II were more likely to suffer from peri-prosthetic joint infection.

While there has been a limited number of case reports of acute gouty attacks after elective TKA in the English scientific literature [38–43], its mechanism remains unclear; one explanation for this could be that the removal of cartilage and synovium during TKA predisposes to the deposition of urate crystals in the joint [39]. Each of the case reports emphasize the difficulties in differentiating acute gouty attacks from septic arthritis, due to their similarity in clinical and biochemical presentation, complicated by false-negative results for bacterial culture due to the use of empirical antibiotics. The possibility of misdiagnosing an acute gouty attack as a post-TKA implant infection could be one of the explanations of our findings in the logistic regression analysis.

### Limitations

This study had several limitations, the most important of which is the limited study population of only 626; combined with the low rates of pooled in-hospital postoperative complications at 8.5% (similar to reported rates in the literature), a larger sample size would provide more statistical power for studying the risks of such complications, especially for subgroup analysis.

It should also be noted that TLC and BMI data were only available in, respectively, 86.6% and 66.8% of the cases reviewed; due to this being a retrospective study, the preoperative TLC, weight, and height measurements were not universally taken or recorded preoperatively in this hospital. This may have induced selection bias to the cases analyzed.

Also, our study population was composed almost solely of Han Chinese people, making it difficult to compare it to the existing studies in the scientific literature, most of which are conducted on Western populations with different demographic characteristics.

## Conclusions

In conclusion, this study evaluated the association between biomarkers of preoperative malnutrition and in-hospital postoperative complications amongst patients undergoing elective TKA. Hypoalbuminemia and obesity class II (BMI ≥ 30 kg/m^2^) were identified as useful and potentially modifiable biomarkers for predicting post-elective TKA peri-prosthetic joint infection, whereas low TLC, overweight, and obesity class I were not significantly associated with such risks.

Patients with BMI ≥ 30 kg/m^2^ should be counseled on the increased risk of postoperative complications, and considerations given for delaying elective surgery until their nutritional status is optimized. The sensitivity and reliability of serum albumin and TLC as biomarkers for predicting post-TKA complications, however, are less firmly established and require further investigations and meta-analysis before a consensus can be reached.

## Data Availability

The datasets used and/or analyzed during the current study are available from the corresponding author on reasonable request.
